# Different Definitions of Developmental Disability and Implications for Outcomes

**DOI:** 10.1001/jamahealthforum.2025.5642

**Published:** 2025-12-19

**Authors:** Ari Ne’eman, Hailey Clark

**Affiliations:** 1 Department of Health Policy and Management, Harvard T.H. Chan School of Public Health, Boston, Massachusetts; 2 Harvard PhD Program in Health Policy, Cambridge, Massachusetts

## Abstract

**Importance:**

Populations identified under varying definitions of developmental disability (DD) differ in notable dimensions. Understanding these crucial differences is necessary when deciding which definition to utilize for policy analysis or service planning.

**Objective:**

To examine the distributive implications of 4 different potential definitions of DD using data from the 2023 Survey of Income and Program Participation (SIPP) and the University of Minnesota's Residential Information Systems Project (RISP).

**Design, Setting, and Participants:**

Cross-sectional study of the 2023 SIPP in the US general population, including institutionalized persons, using supplemental information from 2019 RISP reports. Analyses used person-level weights, and 95% CIs were logit-transformed. The 4 definitions of DD are based on self-reported medical diagnoses of those who answered affirmatively to at least 1 of 18 disability screener questions. The narrowest definition captures cerebral palsy, intellectual disability, and/or autism diagnoses, while the broadest definition also includes epilepsy, learning disability diagnoses, and/or those identified by the SIPP learning and developmental disability screener question.

**Main Outcomes and Measures:**

Outcomes include DD prevalence, utilization of long-term services and supports (LTSS), congregate residential facilities, and income support (Supplemental Security Income/Social Security Disability Insurance), self-reported employment, and impairment characteristics.

**Results:**

In the study population of 2535 children and young adults aged 5 to 21 years (1285 [50.7%] males and 1250 [49.3%] females) and 9701 adults aged 22 years or older (4617 [47.6%] males and 5084 [52.4%] females), childhood and young adulthood prevalence of DD ranged from 4.17% (95% CI, 3.41%-5.09%) using the narrowest definition to 15.93% (95% CI, 14.43%-17.55%) using the broadest definition. For adults, DD prevalence varied from 1.24% (95% CI, 1.01%-1.52%) to 8.10% (95% CI, 7.48%-8.77%). Under the narrowest definition, 20.35% (95% CI, 16.67%-24.89%) of children and young adults with DD and 33.05% (95% CI, 26.87%-40.30%) of adults with DD were eligible for LTSS from a state DD agency and 0.98% (95% CI, 0.80%-1.20%) of children and young adults with DD and 9.82% (95% CI, 7.99%-11.98%) of adults with DD were estimated to be residing in a congregate residential setting. As definitions became more comprehensive, employment increased while LTSS eligibility, congregate setting placement, income support receipt (Supplemental Security Income/Social Security Disability Insurance), and functional impairment acuity decreased.

**Conclusions and Relevance:**

Results of this cross-sectional study of US children and young adults suggest that definition choice substantially affects DD prevalence estimates and the relevance of identified populations for service provision and community integration. A narrower approach may better capture individuals at greatest risk of segregation and who are most likely to utilize publicly funded DD services.

## Key Points

**Question:**

What are the implications of varying approaches to defining developmental disabilities (DDs) on population prevalence, service utilization, and other related outcomes?

**Findings:**

In this cross-sectional study of 2535 children and young adults aged 5 to 21 years and 9701 adults aged 22 years or older from the 2023 Survey of Income and Program Participation (SIPP), childhood and young adulthood DD prevalence ranged between 4.17% and 15.93% and adult DD prevalence varied between 1.24% and 8.10%. A narrow definition was most likely to identify those with higher acuity impairments eligible for publicly funded services and at risk of congregate setting placement and unemployment.

**Meaning:**

The findings suggest that a narrower diagnostic definition of DD better identifies individuals who are likely to be impacted by policy changes to publicly funded services.

## Teaser

This cross-sectional study examines different approaches to defining developmental disabilities using data from the 2023 Survey of Income and Program Participation on population prevalence, service utilization, and other related outcomes.

## Introduction

In 1970, US Congress passed the Developmental Disabilities Act, transforming federal legislation previously focused on intellectual disability (ID) services to encompass a broad range of diagnoses that manifest in childhood. The new construct of developmental disability (DD) was a political creation, established as a result of negotiations between ID and cerebral palsy (CP) advocates in the aftermath of the assassination of President Kennedy, when ID advocates needed help to maintain and grow newly established programs that had been championed by the Kennedy family.^1,2^ Although initially a marriage of convenience to maintain political support after President Kennedy's assassination, DD became an increasingly important clinical concept as many persons who would previously have been diagnosed with ID began to receive other diagnoses, requiring service systems focused on ID to shift eligibility criteria to DD in order to serve the same population.^3^ Developmental disability also became a crucial political coalition, serving as the foundation for largely replacing state institutions for people with DD with a community service system far better financed than its counterparts in mental illness.^4,5,6^

Yet, from the beginning, ambiguity existed as to how to define the boundaries of DD.^7^ The intent was for the term to refer to conditions present from childhood and anticipated to have a substantial lifetime impact.^1^ But conflict swiftly emerged on how to define it. To avoid putting forward a specific list of who was “in or out,” in 1978, Congress amended the Developmental Disabilities Act to a functional definition of DD referring to “severe chronic disability” that manifested before age 22 years, was expected to continue indefinitely, and resulted in substantial functional limitations across multiple listed domains, such as self-care and learning.^7^

The question of how to define DD still presents a challenge for researchers. Depending on what definition is used, DD prevalence estimates in children can vary between 3.1% and 17.3%.^8^ Concern has been raised that the absence of a consistent definition of DD calls into question the credibility of research on this population, since it is unclear to whom it refers.^8^ For adults, most researchers rely on the 1994/1995 National Health Interview Survey on Disability’s (NHIS-D’s) prevalence of 0.79%, produced through a population survey of the noninstitutionalized population asking about both diagnoses and functional impairments.^3,6^ More recent estimates of the adult prevalence of DD have not been produced, as most population surveys do not ask adults about diagnostic status or the full range of functional impairments listed in the Developmental Disabilities Act.

In 2022, the Department of Health and Human Services highlighted the need for developing a standardized definition of DD to permit data collection at the point of care, the development of outcome measures, and effective analysis of Medicaid claims data.^9^ In response to this call, this study examines the distributive implications of 4 different potential definitions of DD using data from the 2023 Survey of Income and Program Participation’s (SIPP’s) disability supplement and the University of Minnesota’s Residential Information Systems Project (RISP).^6,9^ We present prevalence data for 4 plausible DD definitions, updating adult DD prevalence for the first time, to our knowledge, in 30 years and contextualizing each definition with information on service utilization, employment, and impairment frequency.

## Methods

We conducted a retrospective, cross-sectional analysis of the 2023 SIPP, a nationally representative, longitudinal household survey.^10^ This study was deemed not human participant research using the Harvard Longwood Campus IRB Decision Tool and therefore exempt from institutional review board review. The SIPP collects information on household members’ disability status using a series of 16 screener questions on functional impairments and 2 screener questions about specific disabilities. These questions include the 6 core functional impairments commonly used in federal surveys (American Community Survey Disability Questions; known as the ACS-6), 3 child-specific functional impairments, 6 adult-specific functional impairments, a question on having a long-term health condition that limits daily activities, a question on having a learning or developmental disability, and a question on having a mental or emotional condition.

Respondents identified through these screener questions are able to report up to 3 diagnoses that cause their limitations. The 2023 SIPP changed the wording of the medical condition questions to reduce confusion that resulted in prior underreporting of medical conditions.

We constructed 4 definitions of DD from the SIPP’s disability and medical condition questions. We then estimated the prevalence of DD under each definition separately for children and young adults ages 5 to 21 years and adults ages 22 years and older in wave 1, using each individual’s month 12 observation to calculate annual prevalence estimates. Wave 1 respondents are nationally representative of the civilian, noninstitutionalized population, and because they were in their first year of participation in the SIPP, respondents were only exposed to the updated wording of the medical condition questions. This cross-sectional study used the Strengthening the Reporting of Observational Studies in Epidemiology (STROBE) reporting guideline.

### Definitions of DD

We examined 4 increasingly comprehensive definitions of DD. Definition 1 captured respondents who report a diagnosis of CP, ID, or autism spectrum disorder and Asperger syndrome (ASD). Definition 2 added epilepsy. Definition 3 incorporated respondents who indicated having a learning or developmental disability in the associated screener question but who did not list a learning disability (LD), including attention-deficit disorder or attention-deficit hyperactivity disorder, as a diagnosis. Definition 4 incorporates respondents who specified an LD diagnosis. eTable 1 in Supplement 1 shows the mapping of diagnoses to the SIPP medical condition variables.

While the NHIS-D used a functional definition of DD consistent with the Developmental Disabilities Act, we define DD primarily in terms of reported diagnoses, an approach that is more consistent with recent childhood DD prevalence studies.^3,11,12^ This diagnosis-based approach is motivated by data availability and shifting norms in DD research and policy. One advantage of a diagnosis-based approach is that it is far more likely to offer a definition possible to operationalize in a consistent fashion when analyzing administrative claims or electronic health records (EHRs).

### Supplementing SIPP With Data on Persons Residing in Institutions and Other Congregate Residential Settings

The SIPP’s sampling frame is limited to noninstitutionalized persons and thus would not include individuals with DD living in institutions and is unlikely to include individuals residing in other congregate residential facilities. Therefore, we augmented our prevalence estimates with estimates of people with DD living outside of their family home (for children and young adults) and living outside either their family home or their own home (for adults) as well as those living in an intermediate care facility for individuals with intellectual disabilities (for all age groups) from the RISP, an annual survey of state DD service agencies on the number of people with DD residing in different types of residential settings. Although the most recent year for which RISP data are available is 2020, we used 2019 RISP reports to avoid confounding from temporary settings transitions related to the COVID-19 public health emergency and then rescaled the estimates to reflect age group–specific population change between 2019 and 2023.^6,13,14^ eTable 2 in Supplement 1 presents the rescaled SIPP data for children and young adults from birth to 21 years and eTable 3 in Supplement 1 presents these data for adults ages 22 years and older.

### Statistical Analysis

All SIPP prevalence estimates are weighted using the person-level annual weight from the month 12 observation. We calculated 95% CIs using a logit-transformation to ensure that all estimates are bound between 0% and 100%. Analyses used R, version 4.3.1 (R Foundation for Statistical Computing).

We then compared our estimates of the DD population with several different service outcomes: (1) the number of people eligible to receive long-term services and supports (LTSS) from a state DD agency (in RISP: known to or receiving services from a state DD agency), (2) the number of people with DD residing in congregate residential settings, and (3) the number of people receiving income support benefits (Supplemental Security Income [SSI]/Social Security Disability Insurance [SSDI]). Data on the number of persons eligible for LTSS or residing in congregate residential placement come from rescaled RISP reports, while data on income support utilization come from the 2023 SSI and SSDI Annual Statistical Reports (see eTable 4 in Supplement 1 for income support utilization for children and young adults ages 5-21 years and eTable 5 in Supplement 1 for adults 22 years and older).

Because RISP does not disaggregate service settings by recipient diagnosis, we assume that persons eligible for LTSS or residing in congregate residential settings fall into our most narrow definition of DD. Supplemental analyses using National Core Indicators–Intellectual and Developmental Disabilities, a national probability sample of persons receiving LTSS from state DD agencies, found that between 93% and 95% of adults receiving case management and at least 1 paid service from state agencies would be identified under our definition 1, supporting this assumption (eTable 6 in Supplement 1).^15^ For income support receipt, we show SSI and SSDI utilization levels for persons with a diagnosis of ASD or ID in our primary analysis, which we can confirm fit under definition 1. We compared these utilization levels with the self-reported SSI/SSDI rates of those in the SIPP under each DD definition. In a supplementary analysis, we also report utilization levels for persons within the Social Security Administration’s ASD, ID, and DD categories and persons in the ASD, ID, DD, neurocognitive disorders, and childhood disorder not elsewhere classified categories. Finally, we characterized the SIPP populations identified under each of our DD definitions, by examining self-reported employment (compared with persons who do not fit any DD definition), frequency of reporting both the ACS-6 and other functional impairments, and proxy response rate.

## Results

### DD Prevalence

In the study population of 2535 children and young adults aged 5 to 21 years (1285 [50.69%] males and 1250 [49.31%] females) and 9701 adults aged 22 years or older (4617 [47.59%] males and 5084 [52.41%] females), under definition 1 (CP+ID+ASD) we estimated the prevalence of DD among children and young adults ages 5 to 21 years to be 4.17% (95% CI, 3.41%-5.09%) (Table 1). Under definition 2, DD prevalence increases to 4.44% (95% CI, 3.65%-5.38%). Under definition 3, it increases to 7.34% (95% CI, 6.32%-8.2%), and then more than doubles to 15.93% (95% CI, 14.43%-17.55%) under definition 4.

**Table 1.** Estimated DD Prevalence for Children and Young Adults Aged 5 to 21 Years by DD Definition

DD definition	2023 SIPP prevalence, weighted % (95% logit CI)^a^	Estimated population, No. (95% CI)^b^		Prevalence, % (95% CI)
		Identified by SIPP	Identified by SIPP + 2023 estimated RISP	
Definition 1 (CP, ID, ASD)	4.13 (3.37-5.05)	2 959 691 (2 414 805-3 621 105)	2 988 965 (2 444 079-3 650 379)	4.17 (3.41-5.09)
Definition 2 (CP, ID, ASD, EP)	4.40 (3.61-5.34)	3 154 079 (2 592 158-3 831 061)	3 183 352 (2 621 431-3 860 335)	4.44 (3.65-5.38)
Definition 3 (CP, ID, ASD, EP, LD/DD screener, no LD diagnosis)	7.30 (6.28-8.48)	5 238 504 (4 504 444-6 081 366)	5 267 777 (4 533 718-6 110 640)	7.34 (6.32-8.52)
Definition 4 (CP, ID, ASD, EP, LD/DD screener, LD diagnosis)	15.89 (14.39-17.51)	11 396 300 (10 319 592-12 562 367)	11 425 574 (10 348 866-12 591 641)	15.93 (14.43-17.55)

Abbreviations: ASD, autism spectrum disorder and Asperger syndrome; CP, cerebral palsy; DD, developmental disability; EP, epilepsy; ID, intellectual disability; LD, learning disability; RISP, Residential Information Systems Project; SIPP, Survey of Income and Program Participation.

Consistent with prior work, the prevalence of DD is markedly lower among adults ages 22 years and older. Of the adult population, 1.24% (95% CI, 1.01%-1.52%) is estimated to have a DD using definition 1 and 1.51% (95% CI, 1.26%-1.81%) using definition 2 (Table 2). Definition 3 increases the DD prevalence to 4.32% (95% CI, 3.86%-4.82%). Under definition 4, 8.10% (95% CI, 7.48%-8.77%) of the adult population would qualify as having a DD.

**Table 2.** Estimated DD Prevalence for Adults Aged 22 Years or Older by DD Definition

DD definition	2023 SIPP prevalence, weighted % (95% logit CI)^a^	Estimated population, No. (95% CI)^b^		Prevalence, % (95% CI)
		Identified by SIPP	Identified by SIPP + 2023 estimated RISP	
Definition 1 (CP, ID, ASD)	1.12 (0.89-1.40)	2 735 379 (2 183 973-3 424 037)	3 033 371 (2 481 965-3 722 029)	1.24 (1.01-1.52)
Definition 2 (CP, ID, ASD, EP)	1.39 (1.14-1.69)	3 397 830 (2 788 735-4 137 683)	3 695 822 (3 086 727-4 435 675)	1.51 (1.26-1.81)
Definition 3 (CP, ID, ASD, EP, LD/DD screener, no LD diagnosis)	4.20 (3.74-4.70)	10 265 829 (9 151 657-11 509 017)	10 563 821 (9 449 649-11 807 009)	4.32 (3.86-4.82)
Definition 4 (CP, ID, ASD, EP, LD/DD screener, LD diagnosis)	7.98 (7.36-8.65)	19 532 671 (18 015 471-21 165 712)	19 830 663 (18 313 463-21 463 704)	8.10 (7.48-8.77)

Abbreviations: ASD, autism spectrum disorder and Asperger syndrome; CP, cerebral palsy; DD, developmental disability; EP, epilepsy; ID, intellectual disability; LD, learning disability; RISP, Residential Information Systems Project; SIPP, Survey of Income and Program Participation.

^a^ Constructed from a sample of 9701 adults aged 22 years or older.

^b^ In column 3, the estimated population identified by the SIPP was calculated by multiplying the SIPP prevalence (including the 95% logit CI) by the size of the US population aged 22 years or older in 2023 (244 674 386) estimated by the US Census Bureau. The 2019 RISP estimated that 289 269 adults aged 22 years or older were living outside the home or their own home or in an intermediate care facility for individuals with intellectual disabilities. Adjusted to reflect the 2023 adult population, the RISP estimates of the population with intellectual and developmental disabilities living outside the SIPP’s sampling frame added 297 992 adults to the estimated population (including the upper and lower bounds of the 95% CI) presented in column 4.

### Comparing LTSS Utilization and Congregate Residential Placement From External Data Sources

Using definition 1, 20.35% (95% CI, 16.67%-24.89%) of children and young adults ages 5 to 21 years with DD were eligible for LTSS (Table 3), while under definition 4 only 5.32% (95% CI, 4.83%-5.88%) with DD were eligible for LTSS. For adults with DD, 33.05% (95% CI, 26.87%-40.30%) were eligible for LTSS using definition 1. In contrast, using definition 4, 5.06% (95% CI, 4.66%-5.46%) were identified by state DD agencies (Table 4). Our estimate for the proportion of people with DD residing in congregate settings ranged from 0.98% (95% CI, 0.80%-1.20%) (definition 1) to 0.26% (95% CI, 0.23%-0.28%) (definition 4) for children and young adults (Table 3) and between 9.82% (95% CI, 7.99%-11.98%) (definition 1) and 1.50% (95% CI, 1.39%-1.62%) (definition 4) for adults (Table 4).

**Table 3.** Comparison of the Child Population With DD Estimated From SIPP and RISP With Utilization of LTSS, Congregate Residential Settings, and Income Support Programs by Children and Young Adults With DD

DD definition	2023 Estimated population identified by SIPP + 2023 estimated RISP, No. (95% CI)	Estimated % (95% CI)		
		Known or served by state IDD agency	Living in congregate living facility	Receiving SSI (ASD + ID)^a^
Definition 1 (CP, ID, ASD)	2 988 965 (2 444 079-3 650 379)	20.35 (16.67-24.89)	0.98 (0.80-1.20)	14.53 (11.90-17.77)
Definition 2 (CP, ID, ASD, EP)	3 183 352 (2 621 431-3 860 335)	19.11 (15.76-23.21)	0.92 (0.76-1.12)	13.64 (11.25-16.57)
Definition 3 (CP, ID, ASD, EP, LD/DD screener, no LD diagnosis)	5 267 777 (4 533 718-6 110 640)	11.55 (9.96-13.42)	0.56 (0.48, 0.65)	8.25 (7.11-9.58)
Definition 4 (CP, ID, ASD, EP, LD/DD screener, LD diagnosis)	11 425 574 (10 348 866-12 591 641)	5.32 (4.83-5.88)	0.26 (0.23-0.28)	3.80 (3.45-4.20)

Abbreviations: ASD, autism spectrum disorder and Asperger syndrome; CP, cerebral palsy; DD, developmental disability; EP, epilepsy; ID, intellectual disability; IDD, intellectual and developmental disabilities; LD, learning disability; LTSS, long-term services and supports; RISP, Residential Information Systems Project; SIPP, Survey of Income and Program Participation; SSI, Supplemental Security Income.

^a^ The diagnostic groups used to identify those with DD in the SSI data include autism ASD and ID.

**Table 4.** Comparison of the Adult Population With DD Estimated From SIPP and RISP With Utilization of LTSS, Congregate Residential Settings, and Income Support Programs by Adults With DD

DD Definition	2023 Estimated population identified by SIPP + 2023 estimated RISP, No. (95% CI)	Estimated % (95% CI)			
		Known or served by state IDD agency	Living in congregate living facility	Receiving SSI (ASD + ID)^a^	Receiving SSDI (ASD + ID)^a^
Definition 1 (CP, ID, ASD)	3 033 371 (2 481 965-3 722 029)	33.05 (26.87-40.30)	9.82 (7.99-11.98)	29.44 (24.00-35.98)	30.37 (24.75-37.11)
Definition 2 (CP, ID, ASD, EP)	3 695 822 (3 086 727-4 435 675)	27.13 (22.55-32.40)	8.06 (6.70-9.63)	24.17 (20.14-28.93)	24.92 (20.77-29.84)
Definition 3 (CP, ID, ASD, EP, LD/DD screener, no LD diagnosis)	10 563 821 (9 449 649-11 807 009)	9.49 (8.47-10.58)	2.82 (2.52-3.15)	8.45 (7.56-9.45)	8.72 (7.80-9.75)
Definition 4 (CP, ID, ASD, EP, LD/DD screener, LD diagnosis)	19 830 663 (18 313 463-21 463 704)	5.06 (4.66-5.46)	1.50(1.39-1.62)	4.50 (4.16-4.88)	4.64 (4.29-5.03)

Abbreviations: ASD, autism spectrum disorder and Asperger syndrome; CP, cerebral palsy; DD, developmental disability; EP, epilepsy; ID, intellectual disability; IDD, intellectual and developmental disabilities; LD, learning disability; LTSS, long-term services and supports; RISP, Residential Information Systems Project; SIPP, Survey of Income and Program Participation; SSDI, Social Security Disability Insurance; SSI, Supplemental Security Income.

^a^ The diagnostic groups used to identify those with DD in the SSI and SSDI data include autism ASD and ID.

### Comparing Income Support Utilization

When we use ASD and ID as qualifying diagnosis groups for SSI receipt, we calculated that 14.53% (95% CI, 11.90%-17.77%) of children and young adults under definition 1 and 3.80% (95% CI, 3.45%-4.20%) of children and young adults under definition 4 were receiving SSI (Table 3). For adults identified using definition 1, 29.44% (95% CI, 24.00%-35.98%) were receiving SSI and 30.37% (95% CI, 24.75%-37.11%) were receiving SSDI (Table 4). Using definition 4, 4.50% (95% CI, 4.16%-4.88%) of adults with DD were receiving SSI and 4.64% (95% CI, 4.29%-5.03%) of adults with DD were receiving SSDI. Results were substantively similar for other approaches to defining the population of people with DD receiving SSI (see eTables 7 and 8 in Supplement 1) or when relying only on self-reported SSI and SSDI receipt within the SIPP responses (eTables 9 and 10 in Supplement 1).

### Comparing Self-Reported Employment Rates

Employment, measured as reporting a job in the past year, increased when learning disorders were considered in the DD definition. The employment rate for adults with DD under definition 1 was 41.17% (95% CI, 30.51%-52.73%), increasing to 63.44% (95% CI, 59.35%-67.34%) using definition 4 (Figure; eTable 11 in Supplement 1), which is similar to the employment rate for adults without DD under any definition (69.58%; 95% CI, 68.56%-70.57%). We found a similar pattern among children and young adults ages 15 years and older (eTable 12 in Supplement 1).

**Figure.**
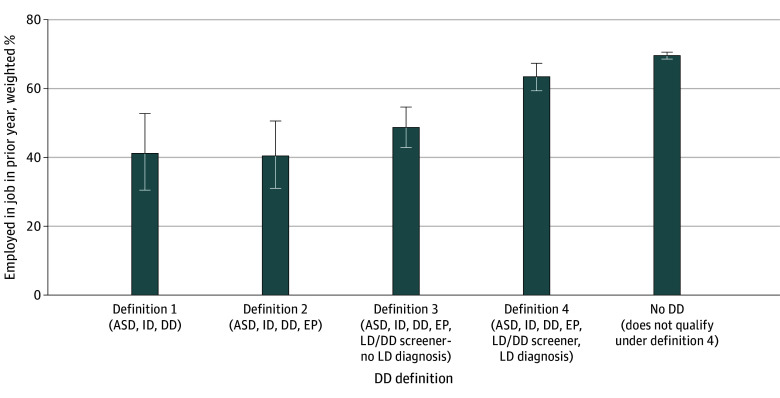
Employment Rate for Adults With Developmental Disability (DD) Aged 22 Years or Older by DD Definition Percentage employed in the prior year for each of the 4 definitions of DD and for the non-DD population. For comparison, we included the employment rate of SIPP respondents aged 22 years or older with no DD, as they did not qualify under our most comprehensive definition 4. Whiskers indicate 95% logit CI. ASD indicates autism spectrum disorder and Asperger syndrome; EP, epilepsy; ID, intellectual disability; LD, learning disability; SIPP, Survey of Income and Program Participation.

### Impairment Characteristics of the DD Population

In eTables 13 to 20 in Supplement 1, we report characteristics of the DD population under each definition using the functional impairment questions within the SIPP. Notably, under definition 1, 71.77% (95% CI, 61.09%-80.45%) of children and young adults with DD reported a cognitive impairment, 23.00% (95% CI, 15.36%-32.96%) of children and young adults reported a self-care impairment, 9.06% (95% CI, 4.58%-17.14%) reported an ambulatory impairment, and 42.19% (95% CI, 25.75%-60.56%) reported an independent living impairment, compared with 59.98% (95% CI, 53.63%-64.13%), 7.93% (95% CI, 5.45%-11.42%), 3.57% (95% CI, 2.04%-6.18%), and 14.87% (95% CI, 9.96%-21.62%) under definition 4, reflecting that, on key dimensions, children and young adults with DD under definition 1 reported far more severe functional impairments than those under definition 4. For adults with DD, definition 4 still yielded substantially lower functional impairment rates, but definitions 2 and 3 yielded comparable or higher impairment rates than definition 1 for cognitive, ambulatory, and independent living impairments. We also found that adults with DD under definition 1 were much more likely to report daily living functional impairments and to provide responses via proxy than under definition 4.

## Discussion

Disability definitions are often best understood as tools for specific purposes. In many contexts, such as applicability of civil rights laws such as the Americans with Disabilities Act, a broad definition of disability is both necessary and appropriate. Similarly, there are important benefits to operationalizing diagnostic categories such as ASD or LD broadly in order to reflect commonalities in disability-related traits that manifest across a range of impairment. In contrast, the concept of DD is closely tied to a system of publicly funded services that emerged for the explicit purpose of supporting persons at the greatest risk of segregation from mainstream community life. The social movement and professional community that developed the concept of DD had as its reason for being bringing people with DD out of institutions and into the broader community, an effort to support community inclusion that remains ongoing in residential, day, and employment service provision.^16,17,18,19^ Our findings suggest that, in so far as continuity is intended with the original purpose of the DD classification, researchers may wish to adopt a more narrow approach.

The functional definition of DD within the Developmental Disabilities Act was intended to focus the definition of DD on this population. Unfortunately, data constraints in both survey and administrative records make it unlikely that a functional definition of DD would prove viable in the analysis of secondary data. The longstanding reliance on the 1994/1995 NHIS-D estimates for adult prevalence of DD illustrates this challenge. This necessitates a shift toward a diagnosis-based approach for calculating prevalence and other research objectives while retaining an emphasis on functional impairment for determining service eligibility, as not every person with a diagnosis of ID, ASD, CP, or any other condition will meet the criteria for DD services. We believe that our updated adult prevalence estimate for DD represents an important contribution to the field, offering researchers and policymakers an estimate that more closely corresponds to current practice in DD and an alternative to reliance on 30-year-old data for DD prevalence in adults.

Our findings also suggest that a diagnosis-based approach can work to operationalize a definition of DD, but the selection of which diagnoses to incorporate has important implications for the relevance of a given DD definition to specific barriers experienced by people with DD. For instance, only 41.17% of adults under our narrow definition 1 are employed, compared with 63.44% under our broadest definition 4, a rate that approaches that of persons without DD under any definition (69.58%). Similarly, while congregate residential placement is a relatively common outcome under definition 1 (9.82%), it is a rare one under definition 4 (1.5%). A definition that corresponds to a narrow approach is likely to make policy changes in state DD agencies directly observable in consumer outcomes, since 20.35% of children and young adults and 33.05% of adults with DD under definition 1 are known to, or served by, state DD agencies. Conversely, such policy changes are unlikely to be detectable under definition 4, where only 5.32% of children and young adults and 5.06% of adults meet this criteria.

Operationalizing a definition of DD for EHR and claims data will require additional work, as the SIPP’s survey-based approach does not permit the same level of granularity as is often present in these other systems. It is likely that other diagnoses will be appropriate to include within the construct of DD that are not present in the SIPP data due to low prevalence, such as spina bifida or spinal muscular atrophy. For example, the Centers for Medicare and Medicaid Services’ Chronic Condition Warehouse includes a broader range of diagnoses in its algorithm for identifying Medicare beneficiaries with “intellectual disabilities and related conditions,” such as fetal alcohol syndrome and Prader-Willi syndrome.^20^ However, these findings offer a valuable starting point and support focusing on those who are most likely to utilize publicly funded supports and who face the greatest risk of segregation from the broader community in future operationalizing of a standard approach for identifying people with DD using diagnostic information.

### Limitations

Our DD definitions are built on self-reported medical conditions rather than administratively documented diagnoses. Consequently, our measures of the DD populations are subject to known limitations associated with self-reporting. Additionally, SIPP respondents were limited to 3 medical conditions, leading to potential underreporting from those with many co-occurring conditions. We augment the SIPP estimates of DD prevalence for children and young adults ages 5 to 21 years with RISP data on children living outside of the family home or in intermediate care facility for individuals with intellectual disabilities for children and young adults from birth to age 21 years. Our underlying assumption is that few children up to age 5 years live outside of the family home and that the impact of the age misalignment on prevalence estimates is minimal. Finally, we used a diagnosis-based approach to articulate each DD definition. Although this is consistent with recent epidemiological practice,^11,12^ it is different from the functional approach articulated within the Developmental Disabilities Act.

## Conclusions

Existing research has used a broad range of possible definitions of DD, with very different implications for prevalence and relevance for publicly funded service provision. These findings suggest that adopting a narrow approach focused on those diagnoses most closely associated with the historical construct of DD, such as ID, autism, and CP, is most likely to identify persons impacted by shifts in publicly funded DD services and at risk of congregate setting placement and exclusion from the workforce. Additional work should seek to operationalize such an approach in administrative and EHR data, addressing the need for a commonly accepted definition of DD for research and policymaking.

## Notes

Supplement 1. **eTable 1.** Mapping of diagnoses to SIPP medical condition variables **eTable 2.** Utilization of LTSS and congregate residential by children from birth to age 21 with DD **eTable 3.** Utilization of LTSS and congregate residential by adults ages 22+ with DD **eTable 4.** Utilization of income support programs by children ages 5-21 with DD **eTable 5.** Utilization of income support programs by adults ages 22+ with DD **eTable 6.** Prevalence of Definition 1 diagnoses in the select years of the National Core Indicators-Intellectual and Developmental Disabilities In-Person Survey (NCI-IDD IPS) **eTable 7.** Comparison of DD child population estimated from SIPP and RISP to utilization of income support programs (alternative definitions) by children with DD **eTable 8.** Comparison of DD adult population estimated from SIPP and RISP to utilization of income support programs (alternative definitions) by adults with DD **eTable 9.** Self-reported SSI receipt by children ages 5-21 with DD **eTable 10.** Self-reported SSI and SSDI receipt by adults with DD **eTable 11.** Self-reported employment rate by adults ages 22+ with DD **eTable 12.** Self-reported employment rate by children ages 15-21 with DD **eTable 13.** Number of ACS-6 functional impairments for children ages 5-21 identified under each DD definition **eTable 14.** Number of ACS-6 functional impairments reported by adults ages 22+ identified under each DD definition **eTable 15.** Prevalence of ACS-6 functional impairments for children ages 5-21 identified under each DD definition **eTable 16.** Prevalence of ACS-6 functional impairments for adults ages 22+ identified under each DD definition **eTable 17.** Prevalence of other disability measures for children ages 5-21 identified under each DD definition **eTable 18.** Prevalence of other disability measures for adults ages 22+ identified under each DD definition **eTable 19.** Proxy response rate for children ages 15-21 with DD **eTable 20.** Proxy response rate for adults ages 22+ with DD

Supplement 2. Data Sharing Statement
