# Supplementary material for: Different Definitions of Developmental Disability and Implications for Outcomes
Source: JAMA Health Forum. 2025 Dec 19;6(12):e255642. doi: 10.1001/jamahealthforum.2025.5642 (PMC12717609; doi:10.1001/jamahealthforum.2025.5642)
Supplement: Supplement 1. — eTable 1. Mapping of diagnoses to SIPP medical condition variables eTable 2. Utilization of LTSS and congregate residential by children from birth to age 21 with DD eTable 3. Utilization of LTSS and congregate residential by adults ages 22+ with DD eTable 4. Utilization of income support programs by children ages 5-21 with DD eTable 5. Utilization of income support programs by adults ages 22+ with DD eTable 6. Prevalence of Definition 1 diagnoses in the select years of the National Core Indicators-Intellectual and Developmental Disabilities In-Person Survey (NCI-IDD IPS) eTable 7. Comparison of DD child population estimated from SIPP and RISP to utilization of income support programs (alternative definitions) by children with DD eTable 8. Comparison of DD adult population estimated from SIPP and RISP to utilization of income support programs (alternative definitions) by adults with DD eTable 9. Self-reported SSI receipt by children ages 5-21 with DD eTable 10. Self-reported SSI and SSDI receipt by adults with DD eTable 11. Self-reported employment rate by adults ages 22+ with DD eTable 12. Self-reported employment rate by children ages 15-21 with DD eTable 13. Number of ACS-6 functional impairments for children ages 5-21 identified under each DD definition eTable 14. Number of ACS-6 functional impairments reported by adults ages 22+ identified under each DD definition eTable 15. Prevalence of ACS-6 functional impairments for children ages 5-21 identified under each DD definition eTable 16. Prevalence of ACS-6 functional impairments for adults ages 22+ identified under each DD definition eTable 17. Prevalence of other disability measures for children ages 5-21 identified under each DD definition eTable 18. Prevalence of other disability measures for adults ages 22+ identified under each DD definition eTable 19. Proxy response rate for children ages 15-21 with DD eTable 20. Proxy response rate for adults ages 22+ with DD [file jamahealthforum-e255642-s001.pdf]

Supplemental Online Content

Ne'eman A, Clark H. Different definitions of developmental disability and their implications. *JAMA Health Forum*. 2025;6(12):e255642. doi:10.1001/jamahealthforum.2025.5642

eTable 1. Mapping of diagnoses to SIPP medical condition variables
eTable 2. Utilization of LTSS and congregate residential by children from birth to age 21 with DD
eTable 3. Utilization of LTSS and congregate residential by adults ages 22+ with DD
eTable 4. Utilization of income support programs by children ages 5-21 with DD
eTable 5. Utilization of income support programs by adults ages 22+ with DD
eTable 6. Prevalence of Definition 1 diagnoses in the select years of the National Core Indicators-Intellectual and Developmental Disabilities In-Person Survey (NCI-IDD IPS)
eTable 7. Comparison of DD child population estimated from SIPP and RISP to utilization of income support programs (alternative definitions) by children with DD
eTable 8. Comparison of DD adult population estimated from SIPP and RISP to utilization of income support programs (alternative definitions) by adults with DD
eTable 9. Self-reported SSI receipt by children ages 5-21 with DD
eTable 10. Self-reported SSI and SSDI receipt by adults with DD
eTable 11. Self-reported employment rate by adults ages 22+ with DD
eTable 12. Self-reported employment rate by children ages 15-21 with DD
eTable 13. Number of ACS-6 functional impairments for children ages 5-21 identified under each DD definition
eTable 14. Number of ACS-6 functional impairments reported by adults ages 22+ identified under each DD definition
eTable 15. Prevalence of ACS-6 functional impairments for children ages 5-21 identified under each DD definition
eTable 16. Prevalence of ACS-6 functional impairments for adults ages 22+ identified under each DD definition
eTable 17. Prevalence of other disability measures for children ages 5-21 identified under each DD definition
eTable 18. Prevalence of other disability measures for adults ages 22+ identified under each DD definition
eTable 19. Proxy response rate for children ages 15-21 with DD
eTable 20. Proxy response rate for adults ages 22+ with DD

This supplemental material has been provided by the authors to give readers additional information about their work.

© 2025 Ne'eman A et al. *JAMA Health Forum*.

Supplemental Material

Supplemental Table 1. Mapping of diagnoses to SIPP medical condition variables

Diagnosis	Medical Condition Variable	Qualifying Value
Cerebral Palsy (CP)	RCONDBRIDGE	Cerebral palsy
Intellectual Disability (ID)	RCONDBRIDGE	Intellectual disability (formerly Mental retardation)
	RCONDSUBTYPE	Neurodevelopmental or neurobehavioral disorders: Intellectual disability (due to a congenital disorder, such as Down Syndrome, or some other cause)
Autism Spectrum Disorder (ASD)	RCONDBRIDGE	Autistic or other developmental disorders
	RCONDSUBTYPE	Neurodevelopmental or neurobehavioral disorders: Autism Spectrum Disorder and Asperger Syndrome
Epilepsy (EP)	RCONDBRIDGE	Epilepsy
	RCONDSUBTYPE	Epilepsy or seizures
Learning Disorder (LD)	RCONDBRIDGE	Learning disability
	RCONDSUBTYPE	Neurodevelopmental or neurobehavioral disorders: Attention Deficit Disorder (ADD) or Attention Deficit-Hyperactivity Disorder (ADHD)<br><br>OR<br><br>Neurodevelopmental or neurobehavioral disorders: Learning disability

Notes: This table presents the technical mapping of diagnoses used to construct the four definitions of developmental disability (DD) to the SIPP medical condition variables. For diagnoses with qualifying values in both the RCONDBRIDGE and RCONDSUBTYPE variables, respondents were classified as having the diagnosis if either variable contained a qualifying value.

Supplemental Table 2. Utilization of LTSS and congregate residential by children from birth to age 21 with DD

	2023 Estimated Population	2023 Estimated Prevalence
Known or Served by State IDD Agency	608,351	0.67%
Living in Congregate Living Facility	29,274	0.03%

Notes: Estimated population known or served by state IDD agency and living in congregate living facility reflects

© 2025 Ne'eman A et al. *JAMA Health Forum*.

estimates from 2019 RISP adjusted to the 2023 population from birth to age 21. 607,899 children from birth to age 21 were known or served by a state IDD agency in 2019, and 29,252 children from birth to age 21 were living in a congregate living facility. The total US population from birth to age 21 was 90,173,422 in 2019 and 90,240,509 in 2023, so we rescale the 2019 values by a factor of 1.0007 to estimate the 2023 populations.

Supplemental Table 3. Utilization of LTSS and congregate residential by adults ages 22+ with DD

	2023 Estimated Population	2023 Estimated Prevalence
Known or Served by State IDD Agency	1,000,219	0.41%
Living in Congregate Living Facility	297,299	0.12%

Notes: Estimated population known or served by state IDD agency and living in congregate living facility reflects estimates from 2019 RISP adjusted to the 2023 population ages 22+. 973,205 adults ages 22+ were known or served by a state IDD agency in 2019, and 289,269 adults ages 22+ were living in a congregate living facility. The total US population ages 22+ was 238,066,101 in 2019 and 244,674,386 in 2023, so we rescale the 2019 values by a factor of 1.0278 to estimate the 2023 populations.

Supplemental Table 4. Utilization of income support programs by children ages 5-21 with DD

	2023 Population	2023 Prevalence
Receiving SSI (ASD + ID)	434,367	0.61%
Receiving SSI (ASD + ID + DD)	634,485	0.88%
Receiving SSI (ASD + ID + DD + NCD + NEC)	675,213	0.94%

Notes: The populations receiving SSI are calculated from the 2023 SSI Annual Statistical Report's number of SSI recipients ages 5 to 21, and the prevalence measures are relative to the total US population ages 5 to 21 in 2023 of 71,729,349. The diagnostic groups used to identify those with DD in the SSI data include: autism spectrum disorders (ASD), intellectual disorders (ID), developmental disorders (DD), neurocognitive disorders (NCD), and childhood disorder not elsewhere classified (NEC).

Supplemental Table 5. Utilization of income support programs by adults ages 22+ with DD

	2023 Population	2023 Prevalence
Receiving SSI (ASD + ID)	893,128	0.48%
Receiving SSI	916,635	0.49%

© 2025 Ne'eman A et al. *JAMA Health Forum*.

(ASD + ID + DD)		
Receiving SSI<br>(ASD + ID + DD + NCD + NEC)	1,037,511	0.56%
Receiving SSDI<br>(ASD + ID)	921,093	0.38%
Receiving SSDI<br>(ASD + ID + DD)	938,976	0.38%
Receiving SSDI<br>(ASD + ID + DD + NCD + NEC)	1,200,449	0.49%

Notes: The populations receiving SSI and SSDI are calculated from the 2023 SSI/SSDI Annual Statistical Report's number of recipients ages 22+, and the prevalence measures are relative to the total US population ages 22+ in 2023 of 244,674,386. The diagnostic groups used to identify those with DD in the SSI data include: autism spectrum disorders (ASD), intellectual disorders (ID), developmental disorders (DD), neurocognitive disorders (NCD), and childhood disorder not elsewhere classified (NEC).

Supplemental Table 6. Prevalence of Definition 1 diagnoses in the select years of the National Core Indicators-Intellectual and Developmental Disabilities In-Person Survey (NCI-IDD IPS)

	% of Respondents with:			
Survey Year	Cerebral Palsy (CP)	Intellectual Disability (ID)	Autism Spectrum Disorder (ASD)	Any Definition 1 Diagnosis (CP, ID, ASD)
2017-2018	16.12%	87.70%	18.47%	94.43%
2018-2019	14.62%	89.66%	19.32%	95.16%
2020-2021	16.64%	83.75%	22.06%	94.38%
2022-2023	14.08%	82.62%	25.52%	93.73%
2023-2024	13.36%	85.39%	26.79%	92.66%

Notes: The National Core Indicators-Intellectual and Developmental Disabilities In-Person Survey (NCI-IDD IPS) is administered to adults (ages 18+) receiving case management and at least one paid service from a state agency. The 2017-2018 survey had a sample of 25,671 respondents in 36 states; the 2018-2019 survey had a sample of 22,009 respondents in 37 states; the 2020-2021 survey (with COVID supplement) had a sample of 19,991 respondents in 26 states; the 2022-2023 survey had a sample of 25,424 respondents in 33 states; and the 2023-2024 survey had a sample of 17,585 respondents in 30 states.

Supplemental Table 7. Comparison of DD child population estimated from SIPP and RISP to utilization of income support programs (alternative definitions) by children with DD

DD Definition	2023 Estimated Population Identified by SIPP + 2023 Estimated RISP	Est. % Receiving SSI (ASD + ID + DD)	Est. % Receiving SSI (ASD + ID + DD + NCD + NEC)

© 2025 Ne'eman A et al. *JAMA Health Forum*.

Definition 1<br><br>(CP, ID, ASD)	2,988,965	21.23%	22.59%
Definition 2<br><br>(CP, ID, ASD, EP)	3,183,352	19.93%	21.215%
Definition 3<br><br>(CP, ID, ASD, EP, LD/DD Screener - No LD Diagnosis)	5,267,777	12.04%	12.82%
Definition 4<br><br>(CP, ID, ASD, EP, LD/DD Screener, LD Diagnosis)	11,425,574	5.55%	5.91%

Notes: Column 2 presents the estimated IDD population size using the SIPP and RISP (Table 1, Column 4). The diagnostic groups used to identify those with DD in the SSI data include: autism spectrum disorders (ASD), intellectual disorders (ID), developmental disorders (DD), neurocognitive disorders (NCD), and childhood disorder not elsewhere classified (NEC).

Supplemental Table 8. Comparison of DD adult population estimated from SIPP and RISP to utilization of income support programs (alternative definitions) by adults with DD

DD Definition	2023 Estimated Population Identified by SIPP + 2023 Estimated RISP	Est. % Receiving SSI (ASD + ID + DD)	Est. % Receiving SSI (ASD + ID + DD + NCD + NEC)	Est. % Receiving SSDI (ASD + ID + DD)	Est. % Receiving SSDI (ASD + ID + DD + NCD + NEC)
Definition 1<br><br>(CP, ID, ASD)	3,033,371	30.22%	34.20%	30.95%	39.57%
Definition 2<br><br>(CP, ID, ASD, EP)	3,695,822	24.80%	28.07%	25.41%	32.48%
Definition 3<br><br>(CP, ID, ASD, EP, LD/DD Screener - No LD Diagnosis)	10,563,821	8.68%	9.82%	8.89%	11.36%

© 2025 Ne’eman A et al. *JAMA Health Forum*.

Definition 4<br><br>(CP, ID, ASD, EP, LD/DD Screener, LD Diagnosis)	19,830,663	4.62%	5.23%	4.73%	6.05%

Notes: Column 2 presents the estimated IDD population size using the SIPP and RISP (Table 2, Column 4). The diagnostic groups used to identify those with DD in the SSI data include: autism spectrum disorders (ASD), intellectual disorders (ID), developmental disorders (DD), neurocognitive disorders (NCD), and childhood disorder not elsewhere classified (NEC).

Supplement Table 9. Self-reported SSI receipt by children ages 5-21 with DD

DD Definition	% Reporting SSI Receipt
Definition 1<br><br>(CP, ID, ASD)	11.90%<br>(6.75%, 20.85%)
Definition 2<br><br>(CP, ID, ASD, EP)	12.10%<br>(6.78%, 20.66%)
Definition 3<br><br>(CP, ID, ASD, EP,<br>LD/DD Screener - No LD Diagnosis)	9.54%<br>(5.82%, 15.24%)
Definition 4<br><br>(CP, ID, ASD, EP,<br>LD/DD Screener, LD Diagnosis)	10.11%<br>(7.10%, 14.20%)

Notes: This table presents the percentage of 2023 SIPP respondents ages 5-21 identified under each DD definition that report receiving SSI income in the current month and whose initial reason for receiving SSI is related to blindness or disability. The prevalence is weighted using person-level survey weights, and the 95% CI in parentheses is logit-transformed.

Supplement Table 10. Self-reported SSI and SSDI receipt by adults with DD

DD Definition	% Reporting SSI Receipt	% Reporting SSDI Receipt
Definition 1	34.87%<br>(24.84%, 46.45%)	27.20%<br>(17.93%, 38.99%)

© 2025 Ne'eman A et al. *JAMA Health Forum*.

(CP, ID, ASD)		
Definition 2<br><br>(CP, ID, ASD, EP)	32.10%<br>(23.45%, 42.17%)	25.41%<br>(17.42%, 35.49%)
Definition 3<br><br>(CP, ID, ASD, EP,<br>LD/DD Screener - No LD Diagnosis)	25.66%<br>(20.74%, 31.29%)	17.47%<br>(13.56%, 22.22%)
Definition 4<br><br>(CP, ID, ASD, EP,<br>LD/DD Screener, LD Diagnosis)	16.21%<br>(13.25%, 19.67%)	11.56%<br>(9.17%, 14.47%)

Notes: This table presents the percentage of 2023 SIPP respondents ages 22+ identified under each DD definition that report receiving SSI income in the current month and whose initial reason for receiving SSI is related to blindness or disability (Column 1) and that report receiving Social Security benefits because they are disabled (Column 2). The prevalence is weighted using person-level survey weights, and the 95% CI in parentheses is logit-transformed.

Supplement Table 11. Self-reported employment rate by adults ages 22+ with DD

DD Definition	% Employed in Past Year
Definition 1<br><br>(CP, ID, ASD)	41.17%<br>(30.51%, 52.73%)
Definition 2<br><br>(CP, ID, ASD, EP)	40.43%<br>(31.03%, 50.58%)
Definition 3<br><br>(CP, ID, ASD, EP,<br>LD/DD Screener - No LD Diagnosis)	48.72%<br>(42.87%, 54.61%)
Definition 4<br><br>(CP, ID, ASD, EP,<br>LD/DD Screener, LD Diagnosis)	63.44%<br>(59.35%, 67.34%)

Notes: This table presents the percentage of 2023 SIPP respondents ages 22+ identified under each DD definition that report having a job in the past year. The prevalence is weighted using person-level survey weights, and the 95% CI in parentheses is logit-transformed.

**Supplement Table 12. Self-reported employment rate by children ages 15-21 with DD**	
DD Definition	% Employed in Past Year
Definition 1<br><br>(CP, ID, ASD)	28.09%<br>(14.03%, 48.29%)
Definition 2<br><br>(CP, ID, ASD, EP)	34.29%<br>(20.01%, 52.12%)
Definition 3<br><br>(CP, ID, ASD, EP,<br>LD/DD Screener - No LD Diagnosis)	43.96%<br>(32.78%, 55.79%)
Definition 4<br><br>(CP, ID, ASD, EP,<br>LD/DD Screener, LD Diagnosis)	46.18%<br>(38.38%, 54.17%)

Notes: This table presents the percentage of 2023 SIPP respondents ages 15-21 identified under each DD definition that report having a job in the past year. The prevalence is weighted using person-level survey weights, and the 95% CI in parentheses is logit-transformed.

**Supplemental Table 13. Number of ACS-6 functional impairments for children ages 5-21 identified under each DD definition**							
DD Definition	% Reporting 0 Impairments	% Reporting 1 Impairment	% Reporting 2 Impairments	% Reporting 3 Impairments	% Reporting 4 Impairments	% Reporting 5 Impairments	% Reporting 6 Impairments*
Definition 1<br><br>(CP, ID, ASD)	22.97% (15.05%, 33.42%)	45.58%, (35.69%, 55.82%)	20.69% (13.28%, 30.77%)	3.35% (1.33%, 8.18%)	5.98% (2.72%, 12.62%)	1.43% (0.20%, 9.43%)	0.00%
Definition 2<br><br>(CP, ID, ASD, EP)	24.99% (17.07%, 35.04%)	45.49% (35.94%, 55.39%)	19.41% (12.44%, 29.01%)	3.15% (1.25%, 7.69%)	5.61% (2.55%, 11.87%)	1.34% (0.19%, 8.88%)	0.00%
Definition 3<br><br>(CP, ID,	31.37% (24.49%, 39.17%)	44.90% (37.41%, 52.63%)	15.42% (10.27%, 22.49%)	3.53% (1.73%, 7.03%)	3.98% (1.92%, 8.09%)	0.81% (0.11%, 5.50%)	0.00%

© 2025 Ne’eman A et al. *JAMA Health Forum*.

ASD, EP, LD/DD Screener - No LD Diagnosis)							
Definition 4<br><br>(CP, ID, ASD, EP, LD/DD Screener, LD Diagnosis)	36.29% (31.33%, 41.57%)	49.14% (43.82%, 54.49%)	10.74% (7.59%, 14.99%)	1.62% (0.80%, 3.27%)	1.83% (0.88%, 3.77%)	0.37% (0.05%, 2.58%)	0.00%

Notes: Each row presents the distribution of the number of ACS-6 functional impairments reported by 2023 SIPP respondents ages 5-21 identified under each DD definition. The prevalence is weighted using person-level survey weights, and the 95% CI in parentheses is logit-transformed. *The independent living impairment is only asked of respondents ages 15+. Therefore, for those ages 5-14, the effective maximum number of impairments to report is 5.

Supplemental Table 14. Number of ACS-6 functional impairments reported by adults ages 22+ identified under each DD definition

DD Definition	% Reporting 0 Impairments	% Reporting 1 Impairment	% Reporting 2 Impairments	% Reporting 3 Impairments	% Reporting 4 Impairments	% Reporting 5 Impairments	% Reporting 6 Impairments
Definition 1<br><br>(CP, ID, ASD)	25.34% (16.65%, 36.56%)	22.85% (14.54%, 34.02%)	24.54% (15.98%, 35.75%)	14.84% (8.81%, 23.91%)	10.39% (5.55%, 18.62%)	1.19% (0.17%, 7.96%)	0.85% (0.12%, 5.81%)
Definition 2<br><br>(CP, ID, ASD, EP)	25.82% (17.91%, 35.70%)	22.55% (15.25%, 32.02%)	23.72% (16.17%, 33.39%)	15.65% (10.11%, 23.43%)	9.19% (5.12%, 15.93%)	0.96% (0.13%, 6.48%)	2.12% (0.49%, 8.71%)
Definition 3<br><br>(CP, ID, ASD, EP, LD/DD Screener - No LD Diagnosis)	22.43% (18.01%, 27.57%)	24.21% (19.31%, 29.89%)	20.58% (16.38%, 25.53%)	17.54% (13.75%, 22.12%)	9.02% (6.27%, 12.82%)	4.52% (2.22% , 9.00%)	1.69% (0.73%, 3.86%)
Definition 4	35.55% (31.66%,	29.15% (25.36%,	15.96% (13.18%,	10.18% (8.01%,	5.65% (4.05%,	2.54% (1.28%,	0.97% (0.44%,

(CP, ID, ASD, EP, LD/DD Screener, LD Diagnosis)	39.63%)	33.25%)	19.19%)	12.86%)	7.83%)	4.98%)	2.11%)

Notes: Each row presents the distribution of the number of ACS-6 functional impairments reported by 2023 SIPP respondents ages 22+ identified under each DD definition. The prevalence is weighted using person-level survey weights, and the 95% CI in parentheses is logit-transformed.

Supplemental Table 15. Prevalence of ACS-6 functional impairments for children ages 5-21 identified under each DD definition

DD Definition	% Reporting Cognitive Impairment	% Reporting Self-Care Impairment	% Reporting Ambulatory Impairment	% Reporting Independent Living Impairment*	% Reporting Hearing Impairment	% Reporting Seeing Impairment
Definition 1<br><br>(CP, ID, ASD)	71.77% (61.09%, 80.45%)	23.00% (15.36%, 32.96%)	9.06% (4.58%, 17.14%)	42.19% (25.75%, 60.56%)	4.86%, 2.22%, 10.33%)	4.65% (1.92%, 10.82%)
Definition 2<br><br>(CP, ID, ASD, EP)	67.34% (57.06%, 76.20%)	22.51% (15.18% ,32.06%)	8.50% (4.30%, 16.13%)	39.43% (24.57%, 56.55%)	4.56% (2.08%, 9.70%)	4.99% (2.22%, 10.85%)
Definition 3<br><br>(CP, ID, ASD, EP, LD/DD Screener - No LD Diagnosis)	62.86% (54.96%, 70.13%)	15.19% (10.40%, 21.64%)	5.66% (3.00%, 10.42%)	25.38% (16.55%, 36.85%)	4.46% (2.40%, 8.13%)	6.50% (3.35%, 12.25%)
Definition 4<br><br>(CP, ID, ASD, EP, LD/DD Screener, LD Diagnosis)	59.98% (53.63%, 64.13%)	7.93% (5.45%, 11.42%)	3.57% (2.04%, 6.18%)	14.87% (9.96%, 21.62%)	2.87% (1.58%, 5.15%)	4.35% (2.53%, 7.38%)

Notes: Each column presents the percentage of 2023 SIPP respondents ages 5-21 identified under each DD definition that report having a specific functional impairment from the set of ACS-6 disability questions. The prevalence is weighted using person-level survey weights, and the 95% CI in parentheses is logit-transformed.
*Prevalence of independent living impairment is only among those ages 15-21.

© 2025 Ne'eman A et al. *JAMA Health Forum*.

**Supplemental Table 16. Prevalence of ACS-6 functional impairments for adults ages 22+ identified under each DD definition**						
DD Definition	% Reporting Cognitive Impairment	% Reporting Self-Care Impairment	% Reporting Ambulatory Impairment	% Reporting Independent Living Impairment	% Reporting Hearing Impairment	% Reporting Seeing Impairment
Definition 1<br><br>(CP, ID, ASD)	55.57% (44.03%, 66.54%)	23.62% (15.68%, 33.95%)	25.20% (17.17%, 35.37%)	48.99% (37.77%, 60.32%)	6.46% (3.14%, 12.84%)	9.22% (4.64%, 17.51%)
Definition 2<br><br>(CP, ID, ASD, EP)	53.72% (43.67%, 63.47%)	21.68% (14.80%, 30.60%)	27.95% (20.35%, 37.06%)	49.05% (39.17%, 59.00%)	8.88% (5.01%, 15.25%)	9.91% (5.44%, 17.37%)
Definition 3<br><br>(CP, ID, ASD, EP, LD/DD Screener - No LD Diagnosis)	63.34% (57.66%, 68.68%)	20.97% (16.44%, 26.37%)	34.11% (28.84%, 39.80%)	42.89% (37.16%, 48.81%)	11.65% (8.87%, 15.15%)	13.88% (10.06%, 18.85%)
Definition 4<br><br>(CP, ID, ASD, EP, LD/DD Screener, LD Diagnosis)	54.28% (50.07%, 58.42%)	12.32% (9.70%, 15.52%)	21.67% (18.43%, 25.29%)	26.60% (23.00%, 30.55%)	9.02% (7.17%, 11.29%)	8.86% (6.62%, 11.76%)

Notes: Each column presents the percentage of 2023 SIPP respondents ages 22+ identified under each DD definition that report having a specific functional impairment from the set of ACS-6 disability questions. The prevalence is weighted using person-level survey weights, and the 95% CI in parentheses is logit-transformed.

**Supplemental Table 17. Prevalence of other disability measures for children ages 5-21 identified under each DD definition**						
	Ages 5-14		Ages 15-21		Ages 5-21	
DD Definition	% Reporting Limited	% Reporting Limitations	% Reporting Health	% Reporting Difficulty	% Reporting Mental or	% Reporting Chronic

© 2025 Ne’eman A et al. *JAMA Health Forum*.

	Ability to Play with Others	with School Work	Condition Limiting Kind or Amount of Work	Finding Job or Remaining Employed	Emotional Condition	Health Condition Limiting Daily Activities
Definition 1<br><br>(CP, ID, ASD)	35.26% (24.61%, 47.60%)	55.09% (42.26%, 67.28%)	61.74% (42.76%, 77.71%)	52.06% (34.23%, 69.38%)	49.72% (39.43%, 60.03%)	52.50% (42.07%, 62.72%)
Definition 2<br><br>(CP, ID, ASD, EP)	36.23% (25.52%, 48.51%)	55.76% (43.00%, 67.81%)	57.81% (40.80%, 73.15%)	44.89% (29.29%, 61.56%)	49.57% (39.64%, 59.54%)	50.20% (40.24%, 60.14%)
Definition 3<br><br>(CP, ID, ASD, EP, LD/DD Screener - No LD Diagnosis)	27.51% (19.60%, 37.14%)	50.24% (39.80%, 60.65%)	41.87% (31.10%, 53.49%)	33.88% (23.91%, 45.52%)	52.06% (44.27%, 59.76%)	43.27% (35.75%, 51.10%)
Definition 4<br><br>(CP, ID, ASD, EP, LD/DD Screener, LD Diagnosis)	18.79% (14.12%, 24.56%)	46.80% (39.64%, 54.09%)	28.22% (21.59%, 35.95%)	21.25% (15.59%, 28.29%)	46.12% (40.80%, 51.52%)	32.01% (27.26%, 37.17%)

Notes: Each column presents the percentage of 2023 SIPP respondents ages 5-21 identified under each DD definition that report having a specific limitation in the set of SIPP disability questions. The columns are grouped by the age range of respondents eligible for each disability question. The prevalence is weighted using person-level survey weights, and the 95% CI in parentheses is logit-transformed.

**Supplemental Table 18. Prevalence of other disability measures for adults ages 22+ identified under each DD definition**				
	Ages 22-70		Ages 22+	
DD Definition	% Reporting Health Condition Limiting Kind or Amount of Work	% Reporting Difficulty Finding Job or Remaining Employed	% Reporting Mental or Emotional Condition	% Reporting Chronic Health Condition Limiting Daily Activities
Definition 1	62.80%	60.27%	57.71%	60.18%

© 2025 Ne'eman A et al. *JAMA Health Forum*.

(CP, ID, ASD)	(51.42%, 72.92%)	(48.63%, 70.85%)	(46.15%, 68.48%)	(48.79%, 70.56%)
Definition 2<br><br>(CP, ID, ASD, EP)	62.26%<br>(52.25%, 71.31%)	60.51%<br>(50.22%, 69.95%)	59.37%<br>(49.28%, 68.72%)	61.70%<br>(51.71%, 70.79%)
Definition 3<br><br>(CP, ID, ASD, EP, LD/DD Screener - No LD Diagnosis)	61.02%<br>(55.26%, 66.48%)	49.91%<br>(43.80%, 56.03%)	66.45%<br>(60.84%, 71.63%)	60.57%<br>(54.75%, 66.10%)
Definition 4<br><br>(CP, ID, ASD, EP, LD/DD Screener, LD Diagnosis)	41.36%<br>(37.25%, 45.61%)	35.56%<br>(31.53%, 39.81%)	59.25%<br>(55.09%, 63.27%)	42.56%<br>(38.43%, 46.80%)

Notes: Each column presents the percentage of 2023 SIPP respondents ages 22+ identified under each DD definition that report having a specific limitation in the set of SIPP disability questions. The columns are grouped by the age range of respondents eligible for each disability question. The prevalence is weighted using person-level survey weights, and the 95% CI in parentheses is logit-transformed.

Supplement Table 19. Proxy response rate for children ages 15-21 with DD

DD Definition	% Proxy Response
Definition 1<br><br>(CP, ID, ASD)	49.21%<br>(31.74%, 66.87%)
Definition 2<br><br>(CP, ID, ASD, EP)	47.01%<br>(31.14%, 63.50%)
Definition 3<br><br>(CP, ID, ASD, EP, LD/DD Screener - No LD Diagnosis)	40.26%<br>(29.66%, 51.86%)
Definition 4<br><br>(CP, ID, ASD, EP, LD/DD Screener, LD Diagnosis)	38.71%<br>(31.35%, 46.62%)

© 2025 Ne’eman A et al. *JAMA Health Forum*.

Notes: This table presents the percentage of 2023 SIPP respondents ages 15-21 identified under each DD definition whose response was provided via proxy. The prevalence is weighted using person-level survey weights, and the 95% CI in parentheses is logit-transformed.

Supplement Table 20. Proxy response rate for adults ages 22+ with DD

DD Definition	% Proxy Response
Definition 1<br><br>(CP, ID, ASD)	41.47%<br>(30.57%, 53.28%)
Definition 2<br><br>(CP, ID, ASD, EP)	40.66%<br>(31.06%, 51.04%)
Definition 3<br><br>(CP, ID, ASD, EP,<br>LD/DD Screener - No LD Diagnosis)	26.49%<br>(21.35%, 32.36%)
Definition 4<br><br>(CP, ID, ASD, EP,<br>LD/DD Screener, LD Diagnosis)	21.52%<br>(18.11%, 25.38%)

Notes: This table presents the percentage of 2023 SIPP respondents ages 15-21 identified under each DD definition whose response was provided via proxy. The prevalence is weighted using person-level survey weights, and the 95% CI in parentheses is logit-transformed.

© 2025 Ne’eman A et al. *JAMA Health Forum*.
