# Supplementary material for: Different Definitions of Developmental Disability and Implications for Outcomes
Source: JAMA Health Forum. 2025 Dec 19;6(12):e255642. doi: 10.1001/jamahealthforum.2025.5642 (PMC12717609; doi:10.1001/jamahealthforum.2025.5642)
Supplement: Supplement 2. — Data Sharing Statement [file jamahealthforum-e255642-s002.pdf]

Data Sharing Statement

Ne’eman. Different Definitions of Developmental Disability and Implications for Outcomes. *JAMA Health Forum*. Published December 19, 2025. doi:10.1001/jamahealthforum.2025.5642

Data
Data available: No

Additional Information
Explanation for why data not available: Data is already publicly available from the Census Bureau.
